# Supplementary material for: Prevalence and Genomic Characterization of Rotavirus A from Domestic Pigs in Zambia: Evidence for Possible Porcine–Human Interspecies Transmission
Source: Pathogens. 2023 Sep 27;12(10):1199. doi: 10.3390/pathogens12101199 (PMC10609906; doi:10.3390/pathogens12101199)
Supplement: Supplementary file 1 [file pathogens-12-01199-s001.zip › pathogens-2585448-supplementary.pdf]

pathogens

MDPI

Supplement Material

Prevalence and Genomic Characterization of Rotavirus A from Domestic Pigs in Zambia: Evidence for Possible Porcine–Human Interspecies Transmission

Joseph Ndebe [1,*,†], Hayato Harima [2,†], Herman Moses Chambaro [3], Michihito Sasaki [4], Junya Yamagishi [5], Annie Kalonda [6], Misheck Shawa [7,8], Yongjin Qiu [9,10], Masahiro Kajihara [7,8], Ayato Takada [1,11,12,13], Hirofumi Sawa [1,11,13,14,15], Ngonda Saasa [1] and Edgar Simulundu [1,16,*]

[1] Department of Disease Control, School of Veterinary Medicine, University of Zambia, Lusaka 10101, Zambia; atakada@czc.hokudai.ac.jp (A.T.); h-sawa@czc.hokudai.ac.jp (H.S.); nsaasa@gmail.com (N.S.)
[2] Laboratory of Veterinary Public Health, Faculty of Agriculture, Tokyo University of Agriculture and Technology, Saiwai-cho 3-5-8, Fuchu 183-8509, Tokyo, Japan; harima@go.tuat.ac.jp (H.H.)
[3] Central Veterinary Research Institute (CVRI), Ministry of Fisheries and Livestock, Lusaka 10101, Zambia; hermcham@gmail.com (H.M.C)
[4] Division of Molecular Pathobiology, International Institute for Zoonosis Control, Hokkaido University, N20 W10, Sapporo 001-0020, Japan; m-sasaki@czc.hokudai.ac.jp
[5] Division of Collaboration and Education, International Institute for Zoonosis Control, Hokkaido University, N20 W10, Sapporo 001-0020, Japan; junya@czc.hokudai.ac.jp
[6] Department of Biomedical Sciences, School of Health Sciences, University of Zambia, Lusaka 10101, Zambia; anniekalonda@gmail.com
[7] Hokudai Center for Zoonosis Control in Zambia, School of Veterinary Medicine, University of Zambia, Lusaka 10101, Zambia; misheckshawa@ymail.com (M.S.); kajihara@czc.hokudai.ac.jp (M.K.)
[8] Division of International Research Promotion, International Institute for Zoonosis Control, Hokkaido University, N20 W10, Sapporo 001-0020, Japan
[9] National Institute of Infectious Diseases, Management Department of Biosafety, Laboratory Animal, and Pathogen Bank, Toyama 1-23-1, Tokyo 162-8640, Japan; qiu-y@niid.go.jp
[10] Department of Virology-I, National Institute of Infectious Diseases, Tokyo 162-8640, Japan
[11] Africa Centre of Excellence for Infectious Diseases of Humans and Animals, School of Veterinary Medicine, University of Zambia, Lusaka 10101, Zambia
[12] Division of Global Epidemiology, International Institute for Zoonosis Control, Hokkaido University, N20 W10, Sapporo 001-0020, Japan
[13] One Health Research Center, Hokkaido University, N18 W9, Sapporo 001-0020, Japan
[14] Hokkaido University, Institute for Vaccine Research and Development (HU-IVReD), N21 W11, Sapporo 001-0020, Japan
[15] Global Virus Network, 725 W Lombard Street, Baltimore, MD 21201, USA
[16] Macha Research Trust, Choma 20100, Zambia
* Correspondence: j.ndebe@yahoo.com (J.N.); esikabala@yahoo.com (E.S.)
† These authors contributed equally to this work.

Citation: Ndebe, J.; Harima, H.; Chambaro, H.M.; Sasaki, M.; Yamagishi, J.; Kalonda, A.; Shawa, M.; Qiu, Y.; Masahiro, K.; Takada, A.; et al. Prevalence and Genomic Characterization of Rotavirus A from Domestic Pigs in Zambia: Evidence for Possible Porcine–Human Interspecies Transmission. *Pathogens* **2023**, *12*, x. https://doi.org/10.3390/xxxxx

Academic Editors: Armanda Bastos and David P. Tchouassi

Received: 15 August 2023
Revised: 21 September 2023
Accepted: 21 September 2023
Published: 27 September 2023

Copyright: © 2023 by the authors. Licensee MDPI, Basel, Switzerland. This article is an open access article distributed under the terms and conditions of the Creative Commons Attribution (CC BY) license (https://creativecommons.org/licenses/by/4.0/).

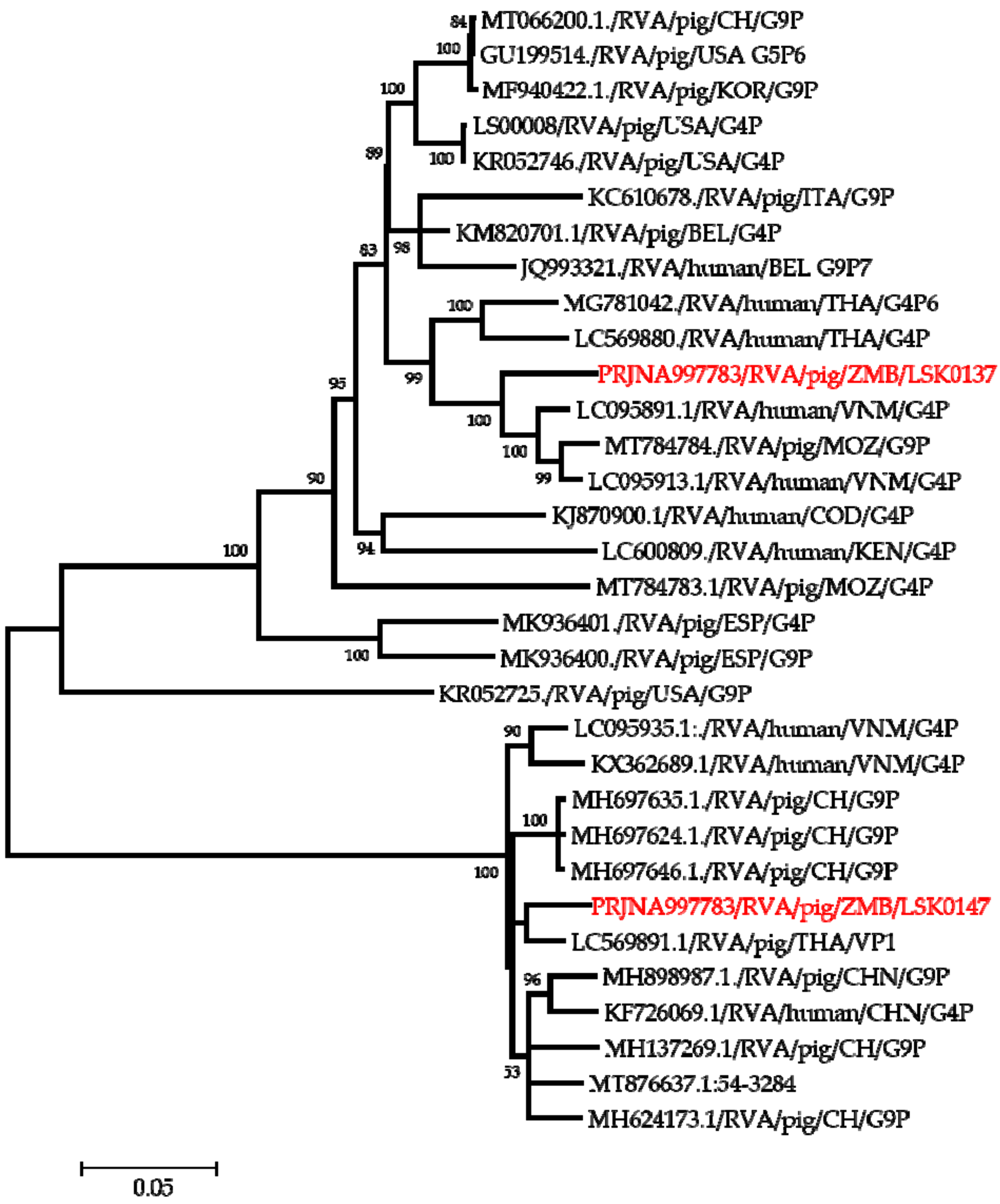

Figure S1. Phylogenetic tree of VP1 genes that belong to genotype R1. The tree was constructed using the general time reversible evolutionary model. The analysis was based on 3218 nucleotides. Bootstrap values on branch nodes ≥ 50% are shown. The GenBank accession number/ Rotavirus group/Species origin/country of origin / G/P-types represent the reference sequences included in the tree. The viruses characterized in this study with their bioproject accession number are shown in red text.

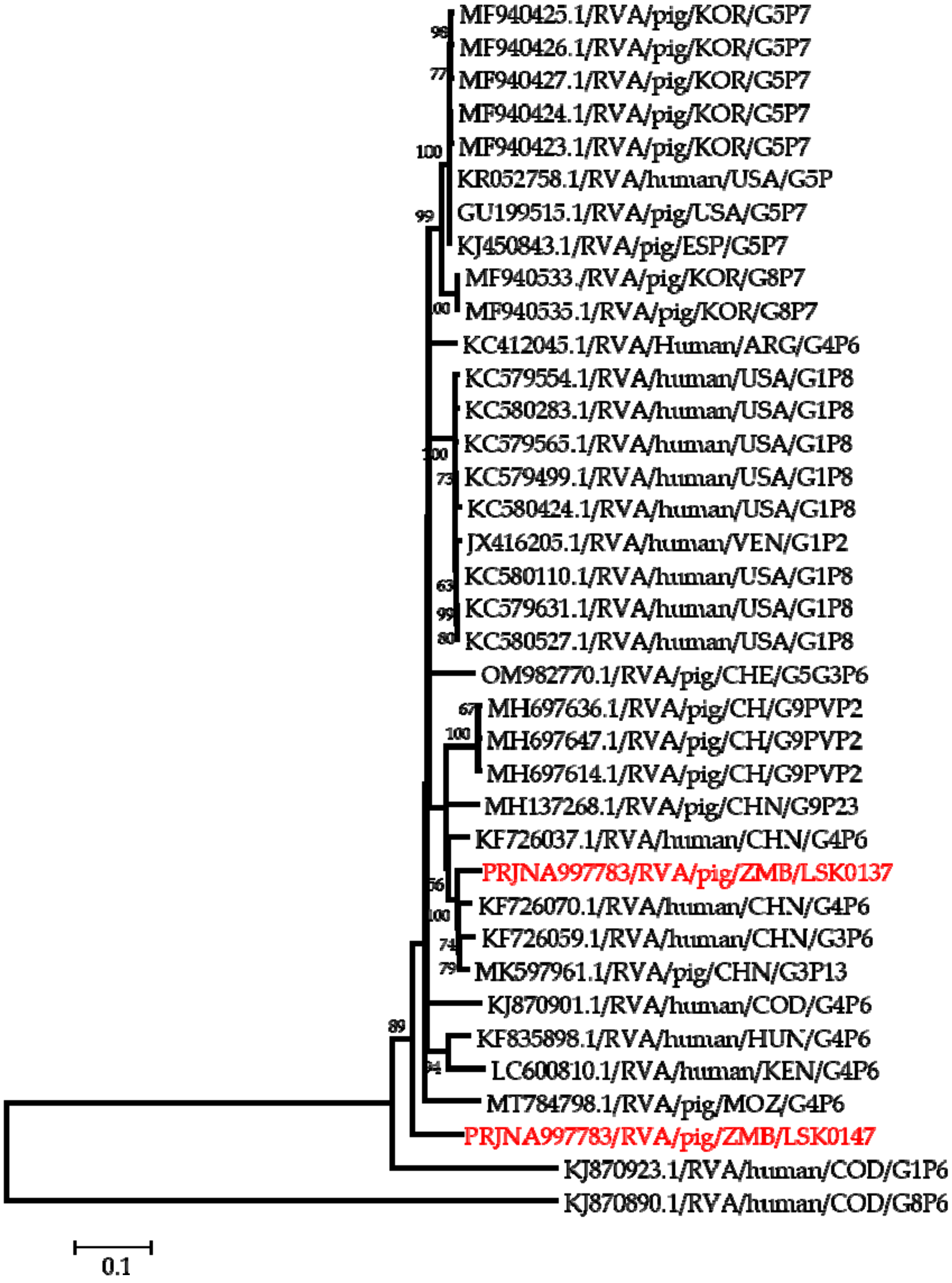

Figure S2. Phylogenetic tree of VP2 genes that belong to genotype C1. The tree was constructed using the general time reversible evolutionary model. The analysis was based on 2605 nucleotides. Bootstrap values on branch nodes ≥ 50% are shown. The GenBank accession number/ Rotavirus group/Species origin/country of origin / G/P-types represent the reference sequences included in the tree. The viruses characterized in this study with their bioproject accession number are shown in red text.

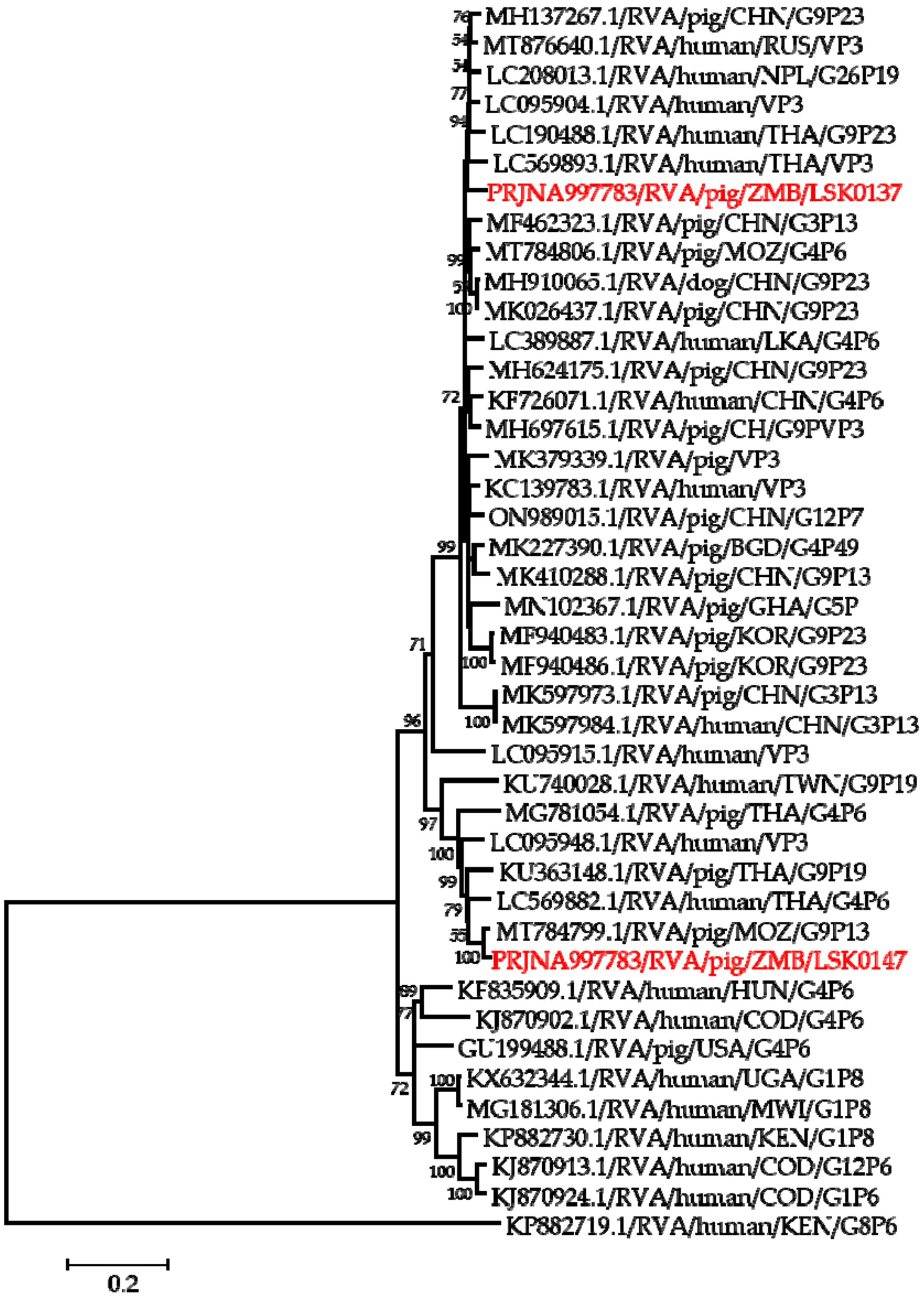

Figure S3. Phylogenetic tree of VP3 genes that belongs to genotype M1. The tree was constructed using the general time reversible evolutionary model. The analysis was based on 2505 nucleotides. Bootstrap values on branch nodes ≥ 50% are shown. The GenBank accession number/ Rotavirus group/Species origin/country of origin / G/P-types represent the reference sequences included in the tree. The viruses characterized in this study with their bioproject accession number are shown in red text.

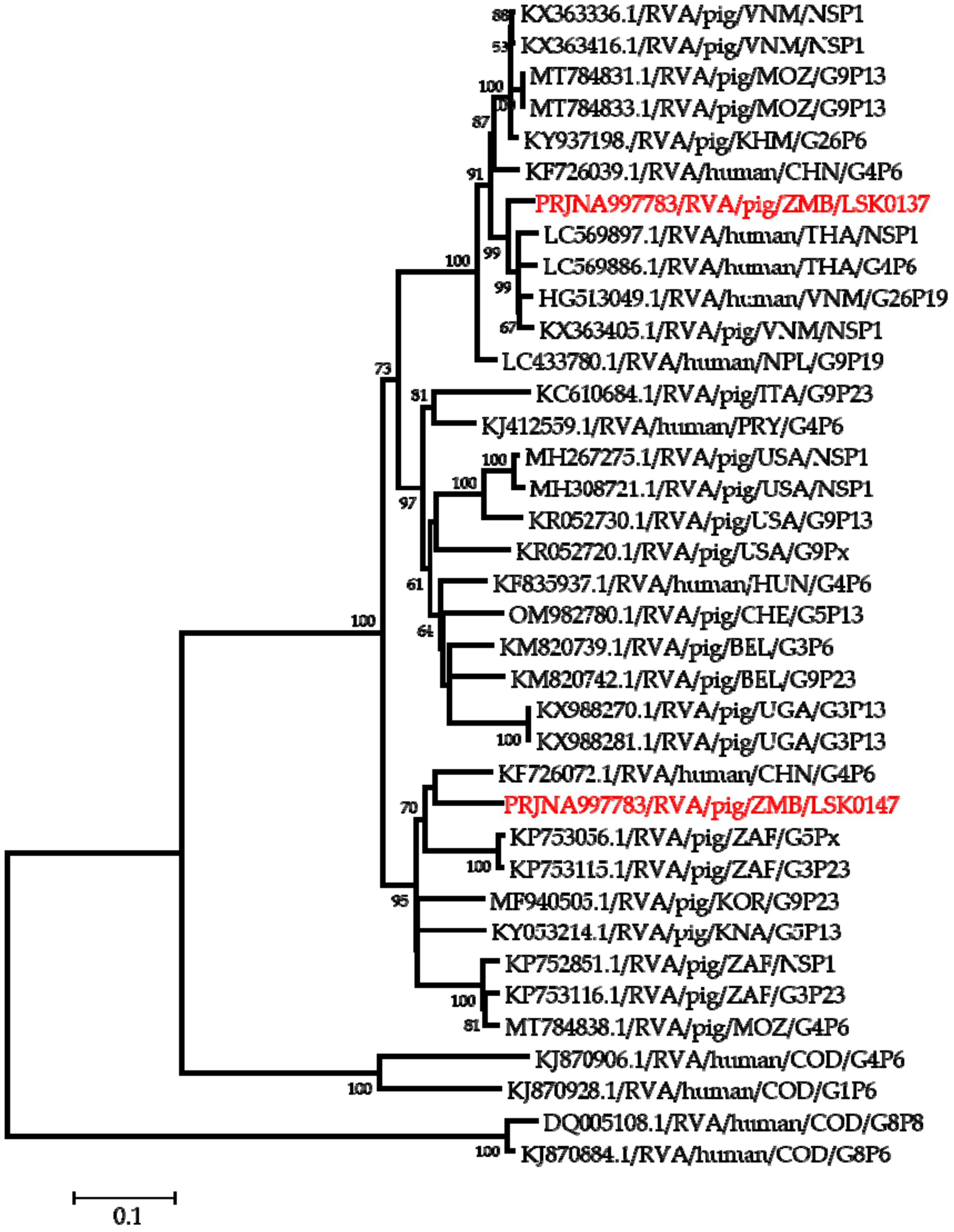

Figure S4. Phylogenetic tree of NSP1 genes. The tree was constructed using the general time reversible evolutionary model. The analysis was based on 1443 nucleotides. Bootstrap values on branch nodes ≥ 50% are shown. The GenBank accession number/ Rotavirus group/Species origin/country of origin / G/P-types represent the reference sequences included in the tree. The virus characterized in this study with its bioproject accession number is shown in red text.

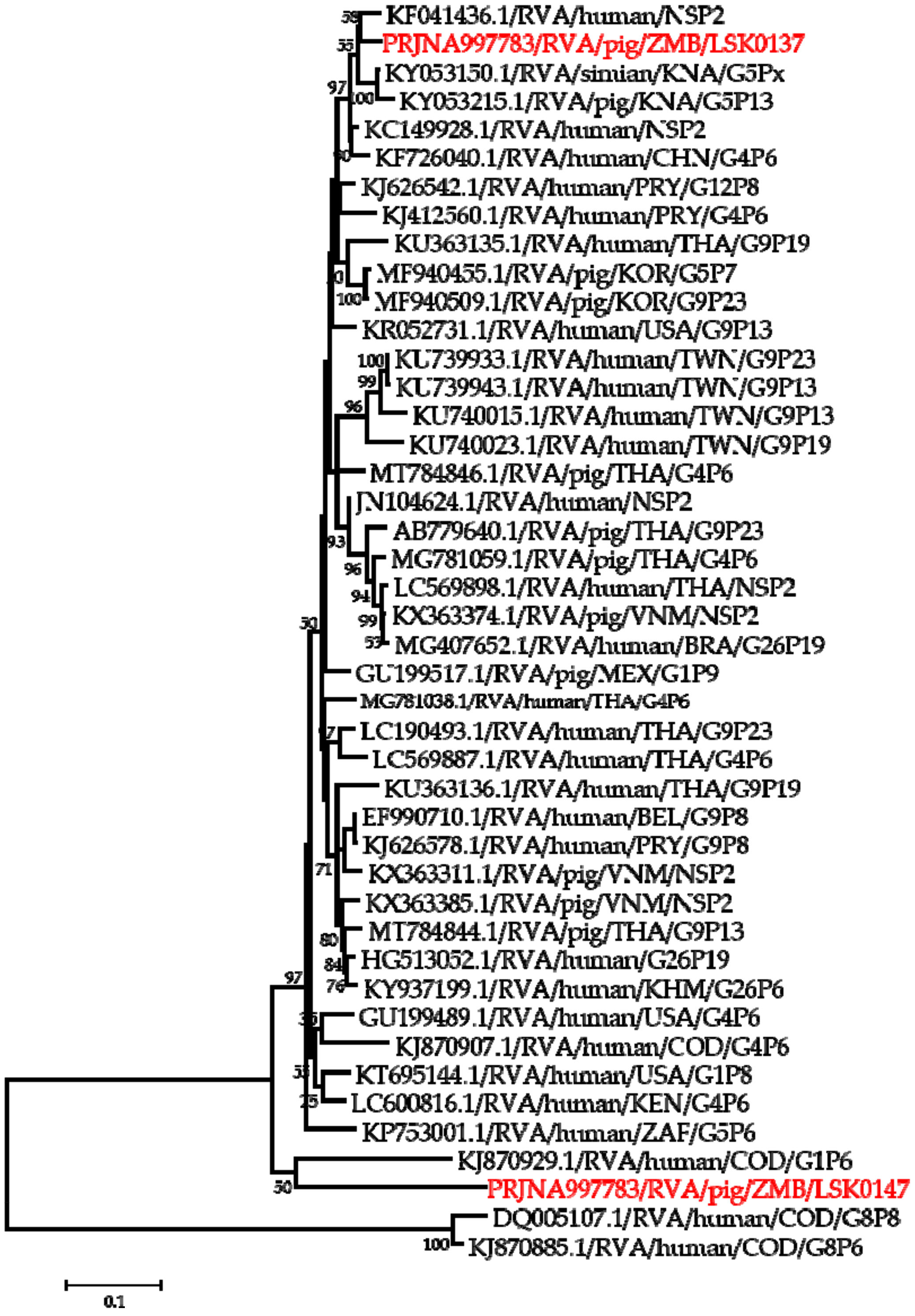

Figure S5. Phylogenetic tree of NSP2 genes that belong to genotype N1. The tree was constructed using the general time reversible evolutionary model. The analysis was based on 804 nucleotides. Bootstrap values on branch nodes ≥ 50% are shown. The GenBank /accession number/ Rotavirus group/Species origin/country of origin / G/P-types represent the reference sequences included in the tree. The viruses characterized in this study with their bioproject accession number are shown in red text.

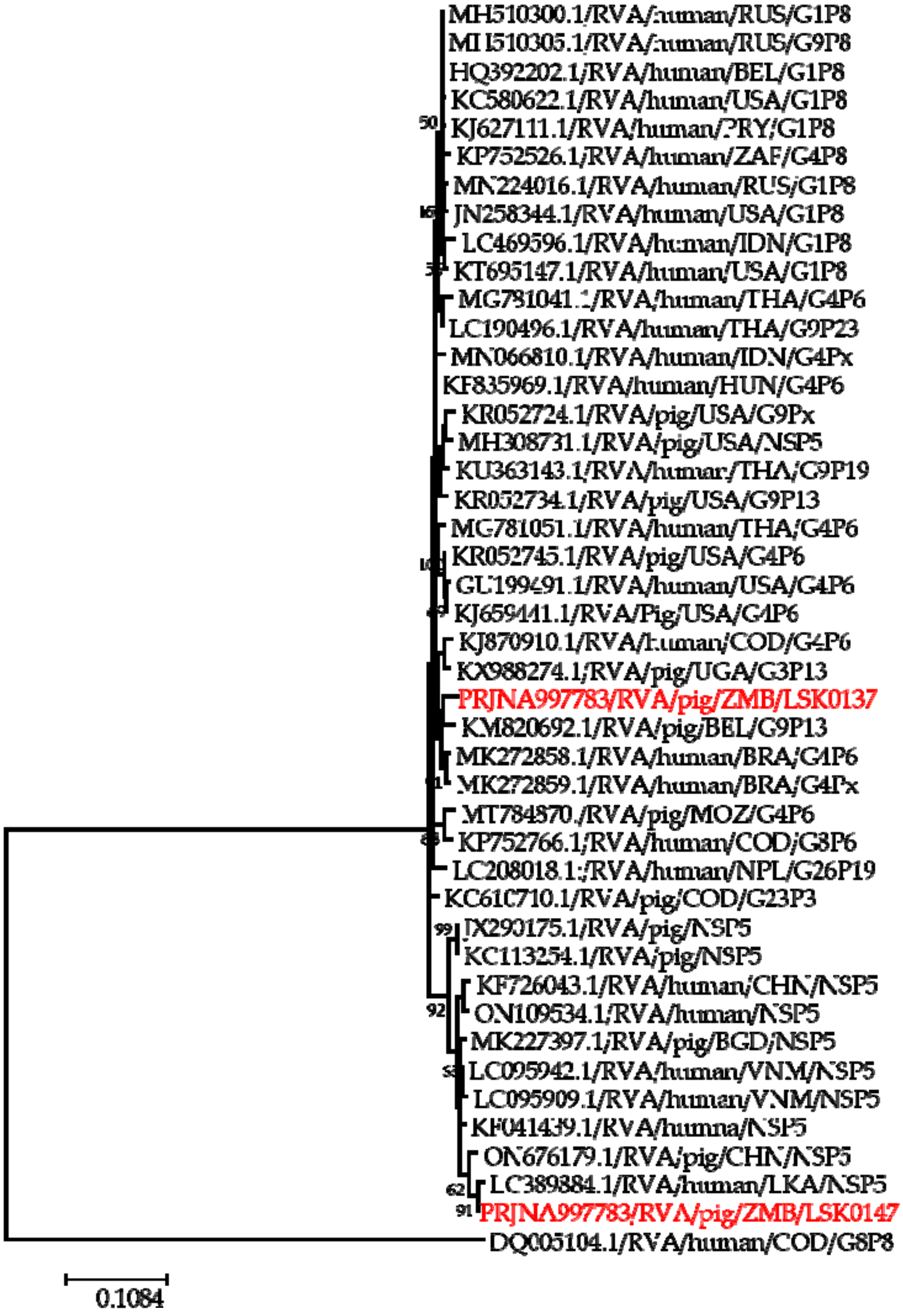

Figure S6. Phylogenetic tree of NSP5 genes that belong to genotype H1. The tree was constructed using the general time reversible evolutionary model. The analysis was based on 582 nucleotides. Bootstrap values on branch nodes ≥ 50% are shown. The GenBank accession number/ Rotavirus group/Species origin/country of origin / G/P-types represent the reference sequences included in the tree. The virus characterized in this study with its bioproject accession number is shown in red text.
